# Three new species of *Oreophryne* (Anura, Microhylidae) from Papua New Guinea

**DOI:** 10.3897/zookeys.333.5795

**Published:** 2013-09-20

**Authors:** Fred Kraus

**Affiliations:** 1 Department of Ecology and Evolutionary Biology, University of Michigan, Ann Arbor, MI 48109, USA

**Keywords:** Adelbert Mts., advertisement calls, Frog, North-coast ranges, Rossel Island, Torricelli Mts

## Abstract

I describe three new species of the diverse microhylid frog genus *Oreophryne* from Papua New Guinea. Two of these occur in two isolated mountain ranges along the northern coast of Papua New Guinea; the third is from Rossel Island in the very southeasternmost part of the country. All three are the first *Oreophryne* known from these areas to have a cartilaginous connection between the procoracoid and scapula, a feature usually seen in species far to the west or from the central cordillera of New Guinea. Each of the new species also differs from the many other Papuan *Oreophryne* in a variety of other morphological, color-pattern, and call features. Advertisement-call data for *Oreophryne* species from the north-coast region suggest that they represent only two of the several call types seen in regions further south, consistent with the relatively recent derivation of these northern regions as accreted island-arc systems. The distinctively different, whinnying, call type of the new species from Rossel Island occurs among other *Oreophryne* from southeastern Papua New Guinea but has been unreported elsewhere, raising the possibility that it may characterize a clade endemic to that region.

## Introduction

Asterophryinae is a subfamily of Microhylidae consisting of 21 genera and hundreds of species that is largely restricted to New Guinea and its satellite islands. Of the constituent genera, *Oreophryne* is presently one of the largest within the Papuan Region, which consists of New Guinea and immediately adjacent islands, the Bismarck and Admiralty Archipelagos, and the Solomon Islands. Currently, the genus has 40 named species in this area (Frost 2013), second in local asterophryine species diversity only to *Cophixalus*, which currently has 42 species (Kraus 2012). Both genera also range outside this area: *Oreophryne* has ten additional species distributed across the Moluccas, Lesser Sundas, Sulawesi, and southern Philippines (Parker 1934, Frost 2013); *Cophixalus* has 18 additional species in northeastern Australia and one more on Halmahera (Parker 1934, Hoskin 2008, 2012, Hoskin and Aland 2011).

Species of *Oreophryne* are usually arboreal dwellers of rainforest habitats, but a few species are terrestrial inhabitants of alpine grasslands (Zweifel et al. 2005, Günther and Richards 2011). The genus is approximately evenly divided into two groups that differ in whether the connection between the procoracoid and scapula consists of a cartilaginous rod or a ligament (Parker 1934). The former group is largely restricted to the central cordillera of New Guinea or areas to the west; *Oreophryne kampeni* is currently the only recognized representative of this group in Papua New Guinea outside of that region, being known only from its type locality near Port Moresby. In contrast, the group having a ligamentous connection is distributed across the entire Papuan range of *Oreophryne* but appears to be absent from the islands west of New Guinea (Parker 1934, Brongersma 1948, Brown and Alcala 1967). There is currently no evidence to suggest if either of these groups is monophyletic. Additional phenotypic features of widespread systematic use for distinguishing among *Oreophryne* species include the presence and degree of toe webbing, relative length of the third vs. fifth toes, size of digital discs, aspects of color pattern, and (more recently) structure of advertisement calls (e.g., Parker 1934, Günther and Richards 2011, Zweifel et al. 2003, 2005).

Several of the early described species of *Oreophryne* have remained uncollected and little studied since their original descriptions, and this long hampered taxonomic study of the genus and diagnosis of new species. Consequently, the genus has received limited taxonomic attention until recently. Nonetheless, of the 40 species of Papuan *Oreophryne*, 24 have been described since 2000, and numerous additional species remain collected but undescribed. Most of these newly described species are from western New Guinea or from the central cordillera (e.g., Günther et al. 2001, Günther 2003a, b), but Zweifel et al. (2003) treated *Oreophryne* from the northern coast region of New Guinea. Herein, I describe a further three distinctive species from Papua New Guinea that belong to the group having a cartilaginous connection between the procoracoid and scapula. Two of these are from some of those same north-coast mountain ranges treated by Zweifel et al. (2003), and the third is from Rossel Island in southeastern-most Papua New Guinea. These bring to four the number of *Oreophryne* from outside the central cordillera of Papua New Guinea that have a cartilaginous connection between the procoracoid and scapula. Other undescribed *Oreophryne* I have from this region have ligamentous connections between the scapulae and procoracoids and will be treated in a later paper.

## Materials and methods

I collected specimens under all applicable institutional animal-care guidelines and provincial and national permits, euthanized them, fixed them in 10% buffered formalin, and then transferred them to 70% ethanol for storage. Livers were removed from representative specimens of each species and stored in 70% ethanol. I made all measurements with digital calipers (SV) or an optical micrometer to the nearest 0.1 mm, with the exception that disc widths were measured to the nearest 0.01 mm. Measurements, terminology, and abbreviations follow Zweifel (1985) and Kraus and Allison (2006): body length from snout to vent (SV); tibia length from heel to outer surface of flexed knee (TL); horizontal diameter of eye (EY); distance from anterior corner of eye to center of naris (EN); internarial distance, between centers of external nares (IN); distance from anterior corner of eye to tip of snout (SN); head width at widest point, typically at the level of the tympana (HW); head length, from tip of snout to posterior margin of tympanum (HL); horizontal tympanum diameter (TY); width of third finger disc (3rdF); and width of the fourth toe disc (4thT). I measured mass to the nearest 0.05 g on freshly euthanized animals using a 10-g Pesola spring scale. I determined sex of animals by examination of vocal slits or by dissection; I determined the cartilaginous nature of the connection between procoracoid and scapula by dissection of alcoholic specimens with a binocular dissecting microscope. Webbing formulae follow Savage and Heyer (1967) as modifed by Myers and Duellman (1982).

I recorded calls in the field using a Sennheiser ME66 microphone with a K6 powering module and either a Sony Professional Walkman WM-D6C cassette recorder, a Sony MDSJE480 minidisc recorder, or a Marantz PMD660 digital audio recorder. I analyzed call structure using the computer program Avisoft-SASLab Pro(v4.34), available from Avisoft Bioacoustics (http://www.avisoft.com/).

I confirmed generic assignment of the frogs using the presence of eleutherognathine maxillae, procoracoids, and clavicles that do not extend to the scapulae (Parker 1934). For discrimination from congeners I relied on direct comparison to museum material (Appendix) as well as to information from Günther (2003a, b), Günther and Richards (2011), Günther et al. (2001, 2009, 2012), Parker (1934), Richards and Iskandar (2000), van Kampen (1913), Zweifel (1956, 2003), and Zweifel et al. (2003, 2005).

Type specimens of new species are deposited in the Bernice P. Bishop Museum, Honolulu (BPBM) and Papua New Guinea National Museum and Art Gallery, Port Moresby (PNGNM). Additional museum abbreviations (Appendix) follow Leviton et al. (1985). Specimens have latitude and longitude coordinates using the Australian Geodetic Datum, 1966 (AGD 66).

## Taxonomy

### Oreophryne cameroni sp. n.

http://zoobank.org/2F75551A-847F-41C0-A6A1-7ACBA408B37E

http://species-id.net/wiki/Oreophryne_cameroni

Figs 1 , 2A, B

#### Holotype.

BPBM 34677 (field tag FK 13704), adult male, collected by F. Kraus at Keki Lodge, Adelbert Mts., 4.7048°S, 145.4042°E, 850 m, Madang Province, Papua New Guinea, 1 October 2009.

#### Paratypes

**(n = 3).** BPBM 34678, same data as holotype, except collected 4 October 2009; BPBM 22689, Siruohu, ~3 km SSE Mt. Sapau summit, Torricelli Mts., 3.3908°S, 142.5297°E, 550–700 m, West Sepik Province, Papua New Guinea; AMNH 78139, Mt. Nibo, 19 km NE Lumi, 700–1550 m, West Sepik Province, Papua New Guinea.

#### Diagnosis.

*Oreophryne cameroni* can be distinguished from all congeners by its unique combination of small size (adult male SV = 19.5–20.4 mm); cartilaginous connection between the scapula and procoracoid; basal toe webbing; fifth toe longer than third; leg moderately long (TL/SV = 0.49–0.51); snout slightly shorter than broad (EN/IN = 0.94–0.95, IN/SV = 0.097–0.103); head relatively broad (HW/SV = 0.37–0.42); finger discs relatively narrow (3rdF/SV = 0.048–0.068); dorsum brown with scattered pustules, white flecks, and darker lateral blotches; venter heavily stippled with brown; dark-brown subocular blotch; dark-brown iris; and call a series of short peeps delivered at a rate of 2.8–2.9 notes/s with a dominant frequency of around 2900 Hz.

#### Comparisons with other species.

The new species differs from all other Papuan *Oreophryne* except *Oreophryne idenburghensis*, *Oreophryne oviprotector*, and *Oreophryne waira* in its unique combination of having a cartilaginous connection between the scapula and procoracoid and basal webbing between the toes. It is easily distinguished from *Oreophryne idenburghensis* by its much smaller size (SV = 19.5–20.4 mm vs. 43–47 mm in *Oreophryne idenburghensis*), longer leg (TL/SV = 0.49–0.51 vs. 0.42–0.44 in *Oreophryne idenburghensis*), broader head (HW/SV = 0.37–0.42 vs. 0.34–0.35 in *Oreophryne idenburghensis*), broader snout (EN/IN = 0.94–0.95 vs. 0.87–0.91 in *Oreophryne idenburghensis*, IN/SV = 0.097–0.103 vs. 0.076–0.081 in *Oreophryne idenburghensis*), and narrower finger discs (3rdF/SV = 0.048–0.068 vs. 0.077–0.091 in *Oreophryne idenburghensis*). It differs from *Oreophryne oviprotector* in its brown dorsal color with scattered white flecks (lime green, without flecks in *Oreophryne oviprotector*), dark-gray ventral coloration (pale translucent gray in *Oreophryne oviprotector*), dark-brown subocular blotch (absent in *Oreophryne oviprotector*), dark-brown iris (coppery brown around pupil and yellowish distally from pupil in *Oreophryne oviprotector*), absence of yellow inguinal and axillary blotches (present in *Oreophryne oviprotector*), absence of conspicuous green bar between eyes (present in *Oreophryne oviprotector*), absence of a white ring around eye (present in *Oreophryne oviprotector*), and call a series of peeps delivered at a rate of 2.8–2.9 notes/s (call a rattle delivered at a rate of 26–28 notes/s in *Oreophryne oviprotector*) and with each note of 71–134 ms duration (each note of approximately 12 ms duration in *Oreophryne oviprotector*). From *Oreophryne waira*, the new species differs in having the fifth toe longer than the third (subequal in *Oreophryne waira*), longer leg (TL/SV = 0.49–0.51 vs. 0.43–0.46 in *Oreophryne waira*), and call a series of peeps of around 2900 Hz and delivered at a rate of 2.8–2.9 notes/s (call a rattle of around 3600 Hz and delivered at a rate of 15–19 notes/s in *Oreophryne waira*).

#### Description of holotype.

**Figure 1.**
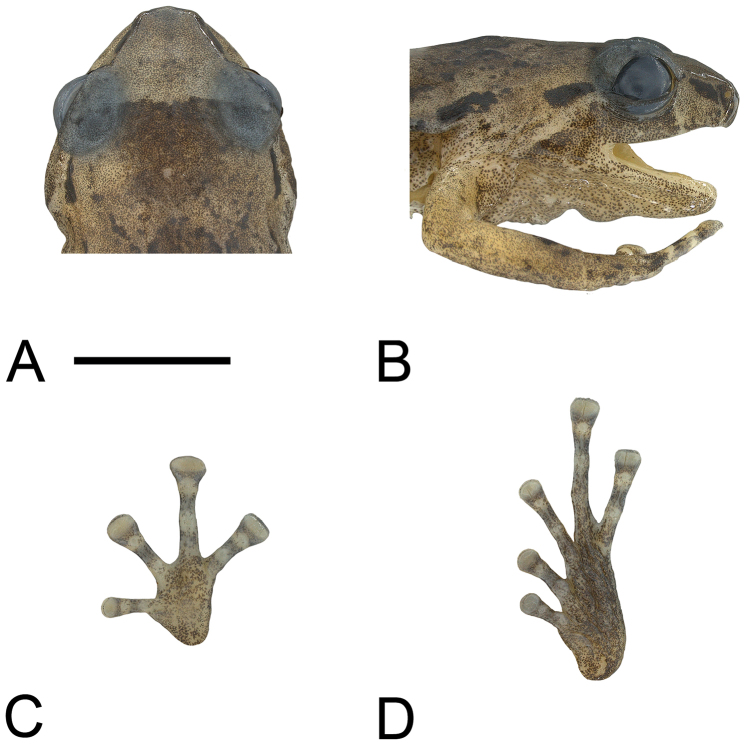
**A** Top of head **B** side of head **C** palmar view of left hand, and **D** plantar view of left foot of holotype of *Oreophryne cameroni* sp. n. (BPBM 34677). Scale bar = 5 mm.

An adult male with an incision on right side and left pectoral region dissected. Procoracoid is connected to the scapula by a cartilaginous rod. Head wide (HW/SV = 0.42), with steep, almost vertical loreal region; upper lip inflated. Canthus rostralis rounded, straight when viewed from above (Fig. 1A). Nostrils directed laterally, much closer to tip of snout than to eyes. Internarial distance slightly wider than distance from naris to eye (EN/IN = 0.95, IN/SV = 0.101, EN/SV = 0.096). Snout slightly rounded when viewed from the side (Fig. 1B), shallowly angulate when viewed from above (Fig. 1A). Eyes moderately large (EY/SV = 0.14); eyelid approximately two-thirds width of interorbital distance. Tympanum small (TY/SV = 0.045), but with a distinct annulus. Skin smooth above and below, except abdomen granular. Supratympanic fold absent. Fingers unwebbed, bearing discs with terminal grooves; relative lengths 3>4>2>1 (Fig. 1C). Finger discs approximately twice width of penultimate phalanges (3rdF/SV = 0.066). Subarticular tubercles low but distinct; inner metacarpal tubercle large, low, oval; outer not apparent. Toes with basal webbing between T2–T5, but absent between T1 and T2; bearing discs with terminal grooves; relative lengths 4>5>3>2>1 (Fig. 1D). Toe discs smaller than those of fingers (4thT/SV = 0.060, 3rdF/4thT = 1.10), somewhat less than twice width of penultimate phalanges. Subarticular tubercles low but distinct; inner metatarsal tubercle a narrow oval; outer not apparent. Hind legs moderately long (TL/SV = 0.51).

In preservative, dorsum dark tan, with a dark-brown smudge between the shoulders, flecked with dark brown dorsally and with a series of darker and larger dashes dorsolaterally extending from behind eye to mid-body. Ground color paler tan on snout and sides; pale region on snout sharply demarcated from darker body color along a front extending between eyes (Fig. 1A). Face pale tan, darker below eye, and with elongate dark-brown blotch on canthus (Fig. 1B). Dorsal surfaces of limbs medium brown; rear of thighs with small pale-straw patch of ground color proximally, distal three-fourths uniform medium brown; front of thighs uniformly medium brown. Ventral surfaces pale straw heavily and uniformly stippled with brown punctations throughout; plantar surfaces more densely stippled with brown. A vaguely defined dark-brown blotch present on each wrist, and a dark-brown ring or blotch present on each finger and toe, each followed distally by a pale-straw blotch at the junction of the last two phalanges. Iris dark brown.

*Measurements of holotype (in mm).*—SV = 19.8, TL = 10.1, HW = 8.4, HL = 7.0, IN = 2.0, EN = 1.9, SN = 3.2, EY = 2.8, TY = 0.9, 3rdF = 1.30, 4thT = 1.18, mass = 0.7 g.

#### Variation.

**Figure 2.**
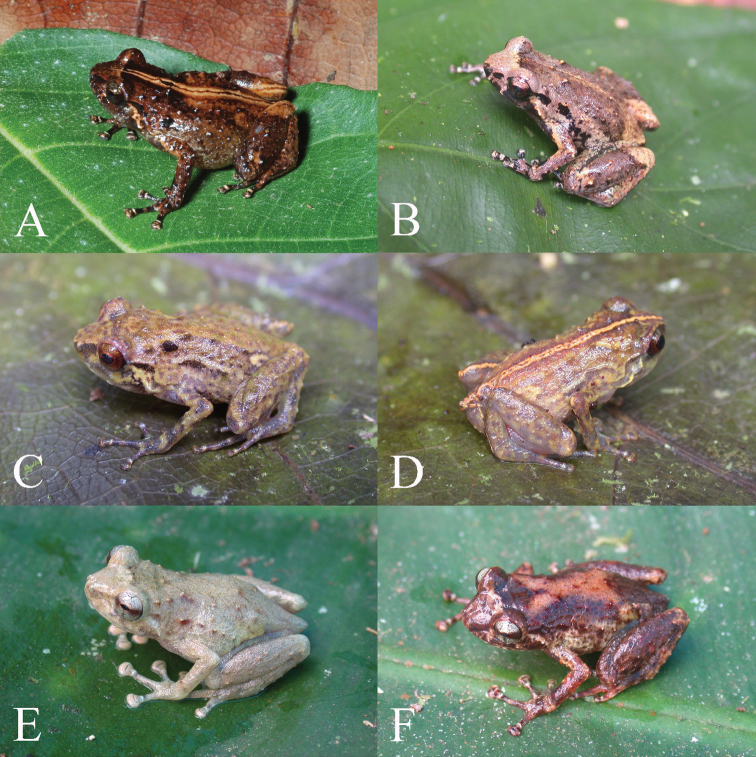
Portraits in life of **A** paratype of *Oreophryne cameroni* sp. n. (BPBM 34678) from Keki Lodge, Adelbert Mts., 850 m elevation **B** paratype of *Oreophryne cameroni* sp. n. (BPBM 22689) from Torricelli Mts., 550 m elevation **C** paratype of *Oreophryne parkopanorum* sp. n. (BPBM 22788) from near summit of Mt. Sapau, Torricelli Mts., 1100–1300 m elevation; and **D** holotype of *Oreophryne parkopanorum* sp. n. (BPBM 22789) from near summit of Mt. Sapau, Torricelli Mts., 1100–1300 m elevation **E** holotype of *Oreophryne gagneorum* sp. n. (BPBM 20542) from Rossel Island, 720 m elevation; and (F) paratype of *Oreophryne gagneorum* sp. n. (BPBM 20544) from Rossel Island, 720 m elevation.

Mensural variation is limited (Table 1), as is to be expected from such a small sample. In life, animals have obvious scattered tubercles (Fig. 2A, B), but these become obscure in preservative. Color pattern shows somewhat more variation. The subadult male from the Torricelli Mts. (BPBM 22689) is similar to the holotype in color pattern except that the dorsum is somewhat darker brown, the brown blotch below the eye is more sharply delimited, and the venter is darker brown overall, with the brown punctations more aggregated and less uniformly dispersed than in the holotype. The second specimen from the Adelbert Mts. (BPBM 34678) is also darker dorsally and laterally than the holotype, and it has an irregular pale-straw stripe mid-dorsally that is broadened into one mid-dorsal pale-straw blotch midway along the back and another one in the sacral region. This pale stripe itself is intermittently bisected by a dark-brown vertebral line. In life, this broadening into blotches along the spine was not evident, and the bisecting brown line was continuous (Fig. 2A). The top of the snout in this specimen is also darker than that in the holotype, but the area between the eyes is pale. The venter is as seen in the holotype. The final specimen (AMNH 78139) also has a broad vertebral stripe on a dark-brown ground color; its venter too is like that seen in the holotype.

**Table 1.** Mensural data for the type series of *Oreophryne cameroni* sp. n. All measurements except mass are in mm.

**Character**	**BPBM 34677**	**BPBM 34678**	**BPBM 22689**	**AMNH 78139**
Sex	M	M	subadult M	M
mass (g)	0.7	–	0.3	–
SV	19.8	20.4	15.6	19.5
TL	10.1	9.9	7.9	9.6
EN	1.9	2.0	1.5	1.8
IN	2.0	2.1	1.6	1.9
SN	3.2	3.1	2.4	2.6
TY	0.9	0.8	0.7	0.9
EY	2.8	2.7	2.1	2.7
HW	8.4	8.0	5.8	7.2
HL	7.0	7.0	5.3	6.4
3rdF	1.30	1.38	0.80	0.94
4thT	1.18	1.15	0.73	0.83
TL/SV	0.51	0.49	0.51	0.49
EN/SV	0.096	0.098	0.096	0.092
IN/SV	0.101	0.103	0.103	0.097
SN/SV	0.16	0.15	0.15	0.13
TY/SV	0.045	0.039	0.045	0.046
EY/SV	0.14	0.13	0.13	0.14
HW/SV	0.42	0.39	0.37	0.37
HL/SV	0.35	0.34	0.34	0.33
3rdF/SV	0.066	0.068	0.051	0.048
4thT/SV	0.060	0.056	0.047	0.043
EN/IN	0.95	0.95	0.94	0.95
3rdF/4thT	1.10	1.20	1.10	1.13
HL/HW	0.83	0.88	0.91	0.89

#### Color in life.

In life, BPBM 34678 was mottled dark brown on a burnt-orange ground dorsally, with the front and rear of thighs the same. An orange-tan vertebral stripe was bisected by a narrow brown vertebral line, the heels were paler than the remainder of the legs, a pale dash extended posteroventrally from the corner of the eye, and white flecks were scattered thoughout the lateral surfaces (Fig. 2A). The venter was pale yellow, heavily stippled with brown, and under the legs was the same. Iris was brown with a greenish cast on the upper half. The subadult animal from the Torricelli Mts. (BPBM 22689) had a paler ground color and greater contrast with the dark brown blotches (Fig. 2B). Field notes for that animal state: “Dorsum mottled tan and brown with black spots on face and sides. Venter light gray heavily flecked with black and silver-gray. Iris brown with upper edge tan.”

#### Call.

I could identify perches of only two calling animals; both called from hidden locations approximately 2.5–3.5 m above ground. I was able to record two calls from the holotype. The call is a short series of relatively rapid peeps.

**Figure 3.**
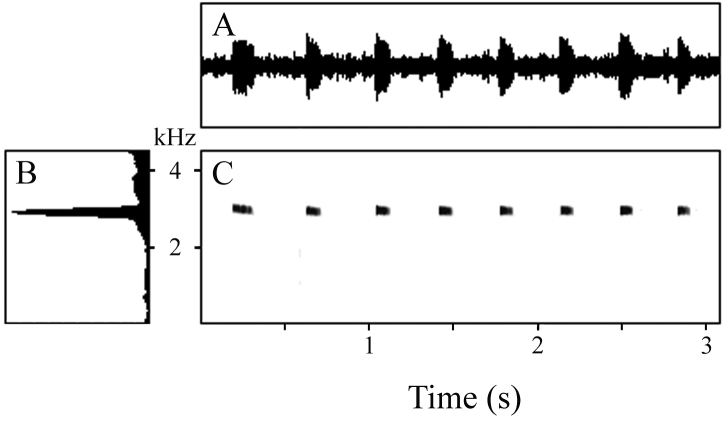
**A** Waveform **B** power spectrum, and **C** spectrogram of 8-note call of the holotype of *Oreophryne cameroni* sp. n. (BPBM 34677) recorded at Keki Lodge, Adelbert Mts., 1 October 2009, air temperature 23.0 °C.

Recorded calls comprised series of 8 and 27 peeps emitted at a rate of 2.64–2.75 notes/s; calls ranged from 2.74–9.55 s in duration (Table 2). Each note was brief, with a mean duration of 0.088 s (range 0.071–0.134 s). The interval between notes was approximately three times longer, averaging 0.280 s and ranging from 0.241–0.389 s. The first 1–3 notes in a series were longer than the remainder; as were the first inter-note intervals. In the second call, the terminal three inter-note intervals were also longer than the preceding intervals. Hence, calls begin somewhat slowly, rapidly reach a regular pace, and may also slow down as they approach termination. There was approximately twice as much variation in inter-note duration than in note duration (Table 2). Notes may have a rounded amplitude envelope or may begin at maximum volume and decrease more or less monotonically, creating either a rounded or an approximately triangular amplitude envelope (Fig. 3A). Notes lack harmonic structure, pulsing, and frequency modulation (Fig. 3C). The dominant frequency of calls varied within a very narrow window (Fig. 3B), averaging 2901 Hz and ranging from 2871–2940 Hz.

**Table 2.** Data for two calls from the holotype of *Oreophryne cameroni* sp. n., BPBM 34677, recorded 1 October 2009, air temperature 23.0 °C. Numbers for call parameters are mean ± SD (range).

**Call series**	**Number of notes**	**Call duration (s)**	**Note duration (s)**	**Inter-note duration (s)**	**Repetition rate (notes/s)**	**Dominant frequency (kHz)**
a	8	2.74	0.101 ± 0.0051<br/> (0.087–0.134)	0.278 ± 0.0091<br/> (0.249–0.316)	2.64	2.906 ± 0.0065<br/> (2.871–2.940)
b	27	9.55	0.084 ± 0.0025<br/> (0.071–0.128)	0.281 ± 0.0071<br/> (0.241–0.389)	2.75	2.900 ± 0.0032<br/> (2.872–2.940)

#### Etymology.

The name is an honorific for my friend H. Don Cameron, professor emeritus of classical studies at University of Michigan and provider of much etymological and grammatical advice on Greek and Latin over the years.

#### Range.

**Figure 4.**
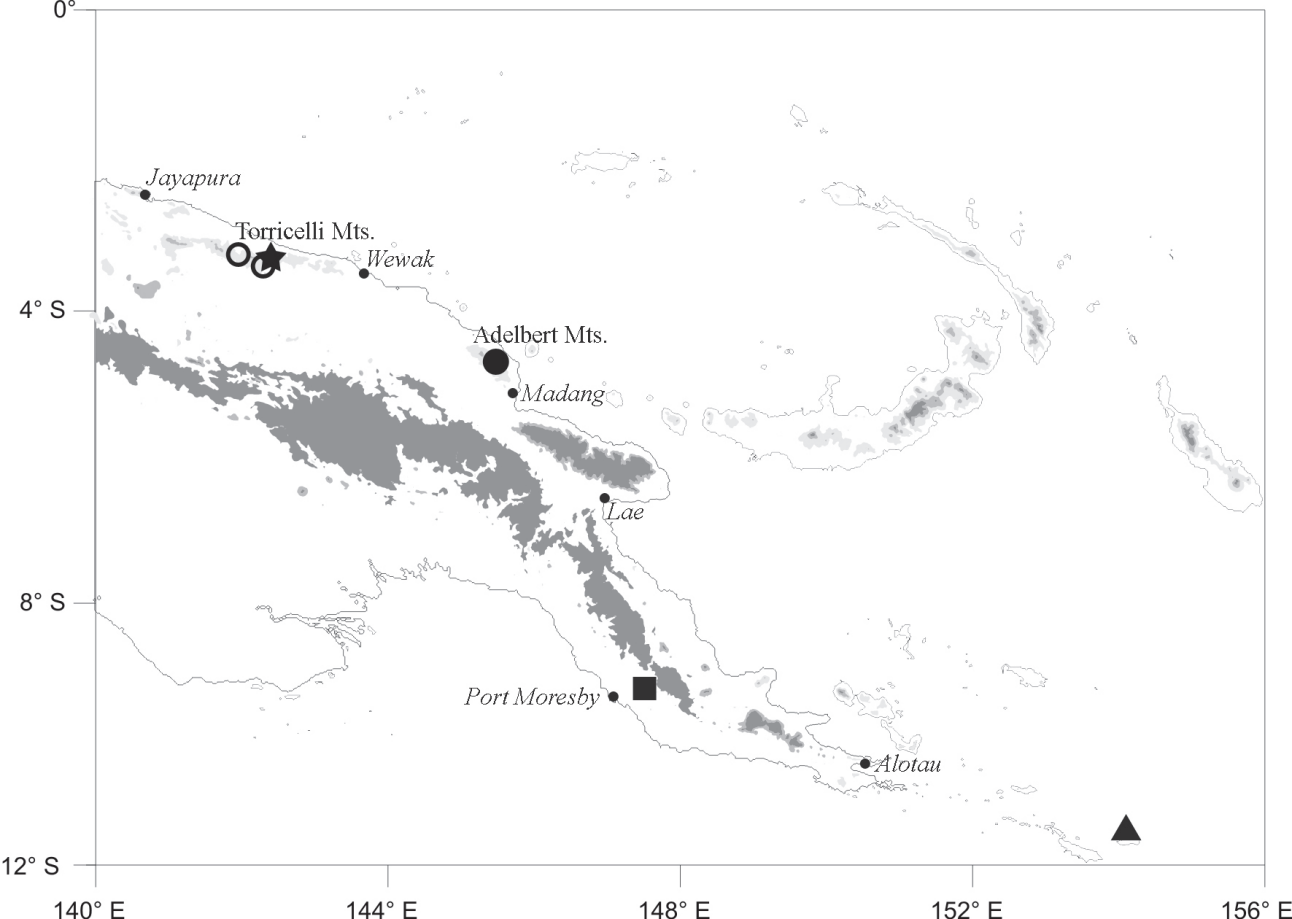
Map of eastern New Guinea showing type locality for *Oreophryne cameroni* sp. n. (filled circle), additional localities for *Oreophryne cameroni* sp. n. (open circles), type locality for *Oreophryne parkopanorum* sp. n. (star), and type locality for *Oreophryne gagneorum* sp. n. (triangle). The square shows the type locality for *Oreophryne kampen* i, the only previous member of the genus with a cartilaginous connection between the scapula and procoracoid known to occur in Papua New Guinea outside the Central Highlands.

Known from the Adelbert Mts., Madang Province, and the Torricelli Mts., West Sepik Province, Papua New Guinea at elevations of 550–850 m (Fig. 4, circles). The species will certainly be found at appropriate elevations in the intervening Prince Alexander Mts. and may, as well, occur in mountain ranges to the west.

#### Ecological notes.

I collected the subadult male in primary lowland rainforest on a steep slope at 550 m elevation in the Torricelli Mts. Canopy was at approximately 30–35 m; understory was rather open and uncrowded; soil was greasy mud. I collected the two calling animals at 850 m in the Adelbert Mts. perched at night in trees approximately 2.5–3.5 m above the ground. These animals were the nearest to the ground that I heard; all others were calling from higher up in the trees. Forest at this site was a clearing edge in remnant primary rainforest on a ridgetop with gentle slopes and rather open understory; soil was thick, sticky clay.

Mature males were 19.5–20.4 mm SV, but one male was still immature at 15.6 mm SV.

Frogs syntopic with this species include *Albericus gudrunae*, *Austrochaperina basipalmata*, *Albericus blumi*, an undescribed *Austrochaperina*, *Callulops microtis*, *Callulops personatus*, *Choerophryne proboscidea*, *Callulops rostellifer*, *Cophixalus balbus*, *Callulops cheesmanae*, *Callulops pipilans*, *Copiula fistulans*, *Copiula tyleri*, *Hylarana arfaki*, *Hylarana garritor*, *Hylarana jimiensis*, *Hylarana papua*, *Hylarana volkerjane*, *Hylophorbus macrops*, *Hylarana proekes*, two undescribed *Hylophorbus*, *Liophryne schlaginhaufeni*, *Litoria arfakiana*, *Litoria genimaculata*, *Litoria wollastoni*, *Oreophryne biroi*, *Mantophryne lateralis*, *Nyctimystes fluviatilis*, *Nyctimystes pulcher*, *Platymantis papuensis*, *Sphenophryne cornuta*, *Xenorhina obesa*, *Xenorhina oxycephala*, and *Xenorhina tumulus*.

#### Remarks.

In their revision of *Oreophryne* species from the northern coast of New Guinea, Zweifel et al. (2003) pointed out that AMNH 78139 was problematic in its identification, not clearly fitting with the other species discussed. In assigning the specimen to this new species, that problem is resolved.

### Oreophryne parkopanorum sp. n.

http://zoobank.org/552EF19B-8CA3-4DD0-9271-DEB337E78FA9

http://species-id.net/wiki/Oreophryne_parkopanorum

Figs 2C, D , 5

#### Holotype.

BPBM 22789 (field tag FK 11847), adult female, collected by F. Kraus 1.2 km S Mt. Sapau summit, Torricelli Mts., 3.3773°S, 142.5180°E, 1120–1320 m, West Sepik Province, Papua New Guinea, 27 May 2005.

#### Paratypes

**(n = 4).** BPBM 22787–88, PNGNM 24152, same data as holotype; BPBM 22790, 1.6 km SSW Mt. Sapau summit, Torricelli Mts., 3.3807°S, 142.5155°E, 1050 m, West Sepik Province, Papua New Guinea.

#### Diagnosis.

*Oreophryne parkopanorum* can be distinguished from all congeners by its unique combination of small size (adult male SV = 17.5–17.7 mm, adult female SV = 20.1 mm); cartilaginous connection between the scapula and procoracoid; unwebbed toes; third toe longer than fifth; leg moderately long (TL/SV = 0.45–0.51); head short (HL/SV = 0.35–0.36, HL/HW = 0.89–0.91), snout long and broad (EN/IN = 0.76–0.89, EN/SV = 0.085–0.097, IN/SV = 0.102–0.120); eye large (EY/SV = 0.14–0.15); finger and toe discs broad (3rdF/SV = 0.048–0.068, 4thT/SV = 0.044–0.053, 3rdF/4thT = 1.08–1.30); longitudinal rows of ridges or pustules on dorsum; dorsum paler mid-dorsally than dorsolaterally; and coppery-brown iris.

#### Comparisons with other species.

The new species differs from all other Papuan *Oreophryne* except *Oreophryne alticola* and *Oreophryne habbemensis* in its unique combination of having a cartilaginous connection between the scapula and procoracoid, absence of toe webbing, and third toe longer than the fifth. *Oreophryne parkopanorum* differs from these species in its longer leg (TL/SV = 0.45–0.51 vs. 0.33–0.38 in *Oreophryne alticola* and *Oreophryne habbemensis*), longer and wider snout (EN/SV = 0.085–0.097 vs. 0.064–0.065 in *Oreophryne alticola*, 0.073–0.081 in *Oreophryne habbemensis*; IN/SV = 0.102–0.120 vs. 0.079–0.088 in *Oreophryne alticola*, 0.082–0.089 in *Oreophryne habbemensis*), larger eye (EY/SV = 0.14–0.15 vs. 0.10–0.13 in *Oreophryne alticola*, 0.12–0.13 in *Oreophryne habbemensis*), broader finger discs (3rdF/SV = 0.048–0.068 vs. 0.031–0.040 in *Oreophryne alticola*, 0.034–0.044 in *Oreophryne habbemensis*), and broader toe discs (4thT/SV = 0.044–0.053 vs. 0.026–0.033 in *Oreophryne alticola*, 0.034–0.038 in *Oreophryne habbemensis*).

Several other species also share with *Oreoprhyne parkopanorum* the combination of a cartilaginous connection between the scapula and procoracoid and absence of toe webbing. Of these, *Oreophryne anamiatoi*, *Oreophryne asplenicola*, *Oreophryne flava*, *Oreophryne graminus*, *Oreophryne notata*, *Oreophryne pseudasplenicola*, and *Oreophryne streiffeleri* are easily distinguished from *Oreoprhyne parkopanorum* in having the fifth toe obviously longer than the third. *Oreophryne brevicrus*, *Oreophryne clamata*, *Oreophryne geminus*, and *Oreophryne terrestris* are somewhat less distinct in this respect in having the third toe subequal to the fifth, instead of distinctly longer. Besides relative toe length, *Oreophryne parkopanorum* further differs from *Oreophryne clamata* in its broader snout (IN/SV = 0.102–0.120 vs. 0.091–0.103 in *Oreophryne clamata*), lesser relative size of finger discs to toe discs (3rdF/4thT = 1.08–1.30 vs. 1.50–1.63 in *Oreophryne clamata*), longer head (HL/SV = 0.35–0.36 vs. 0.28–0.30, HL/HW = 0.89–0.91 vs. 0.70–0.82 in *Oreophryne clamata*), and absence of a dark subocular blotch and black spots around arm insertion (both present in *Oreophryne clamata*).

*Oreophryne brevicrus*, *Oreophryne geminus*, and *Oreophryne terrestris* are all alpine species instead of mid-elevation forest dwellers. Beyond relative toe length, *Oreoprhyne parkopanorum* also differs from *Oreophryne brevicrus* in its longer leg (TL/SV = 0.45–0.51 vs. 0.36–0.42 in *Oreophryne brevicrus*), mid-dorsum paler than dorsolateral regions (mid-dorsum darker than dorsolateral regions in *Oreophryne brevicrus*), and venter with scattered large brown flecks (venter evenly stippled with brown in *Oreophryne brevicrus*); and it differs from *Oreophryne geminus* and *Oreophryne terrestris* in its longer leg (TL/SV = 0.45–0.51 vs. 0.32–0.39 in *Oreophryne geminus*, 0.34–0.44 in *Oreophryne terrestris*), broader finger discs (3rdF/SV = 0.048–0.068 vs. 0.030–0.041 in *Oreophryne geminus*, 0.031–0.042 in *Oreophryne terrestris*), and broader toe discs (4thT/SV = 0.044–0.053 vs. 0.025–0.039 in *Oreophryne geminus*, 0.024–0.042 in *Oreophryne terrestris*).

#### Description of holotype.

**Figure 5.**
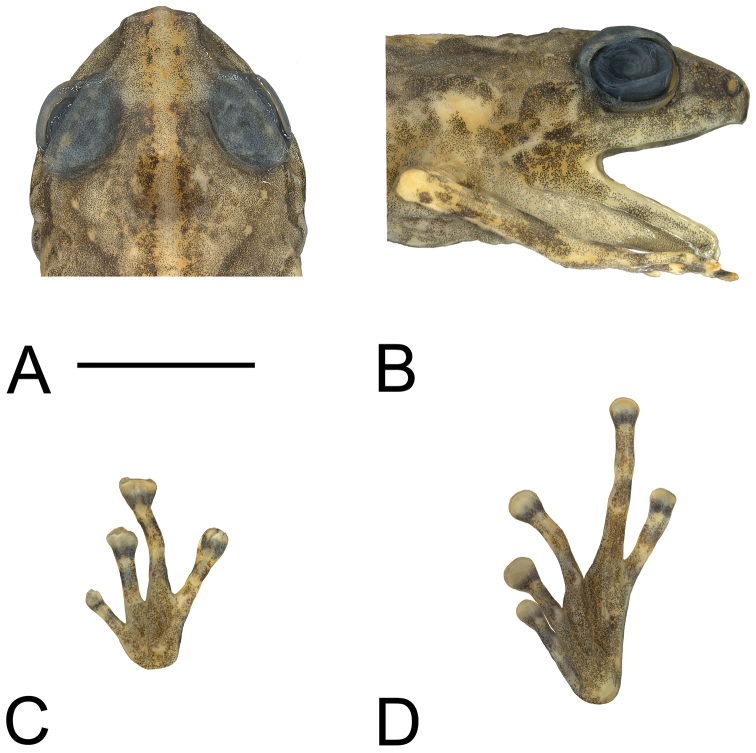
**A** Top of head **B** side of head **C** palmar view of left hand, and **D** plantar view of left foot of holotype of *Oreophryne parkopanorum* sp. n. (BPBM 22789). Scale bar = 5 mm.

An adult female with lateral incision on right side and left pectoral region dissected. Head wide (HW/SV = 0.40), with steeply oblique, slightly concave loreal region; upper lip somewhat inflated. Canthus rostralis rounded, slightly concave when viewed from above (Fig. 5A). Nostrils directed laterally, closer to tip of snout than to eyes. Internarial distance wider than distance from naris to eye (EN/IN = 0.77, IN/SV = 0.109, EN/SV = 0.085). Snout truncate when viewed from the side (Fig. 5B), shallowly angulate when viewed from above (Fig. 5A). Eyes moderately large (EY/SV = 0.14); eyelid approximately two-thirds width of interorbital distance. Tympanum small (TY/SV = 0.050), with distinct annulus, partly covered by surrounding flesh dorsally, projecting ventrally. Dorsum with many raised ridges and series of tubercles, one paired series of tubercles forming an hourglass pattern from behind eyes to posterior of body, each line of warts constricting medially at shoulder and then diverging slightly laterally past this; sides tuberculate; ventral surfaces smooth anteriorly, granular on abdomen. Supratympanic fold absent; few tubercles posterior to tympanum. Fingers unwebbed, bearing discs with terminal grooves; relative lengths 3>4>2>1 (Fig. 5C). Finger discs slightly less than twice width of penultimate phalanges (3rdF/SV = 0.057), except for F1, which is only slightly wider than penultimate phalanx. Subarticular tubercles not obvious; inner metacarpal tubercle oval but low; outer not apparent. Toes unwebbed, bearing discs with terminal grooves; relative lengths 4>3>5>2>1 (Fig. 5D). Toe discs smaller than those of fingers (4thT/SV = 0.044, 3rdF/4thT = 1.32), somewhat less than 1.5 times width of penultimate phalanges on T4 and T5 but wider on T2 and T3. Subarticular tubercles very low or absent; inner metatarsal tubercle large, oval; outer absent. Hind legs moderately long (TL/SV = 0.50).

In preservative, dorsum with pale straw-yellow ground, heavily dusted with brown punctations, with areas having darker dusting and areas lacking dusting arrayed in rows. Pale straw-yellow vertebral stripe; pale straw-yellow blotch above each forearm insertion; pale straw-yellow triangle on top of snout (Fig. 5A). Series of darker-brown flecks dorsolaterally; dark-brown flecks widely scattered laterally; two dark-brown dashes behind eye, one largely superior to the tympanum, the other inferior to it and ending in a brown patch at rictus (Fig. 5B). Face irregularly dusted/mottled with brown, but not as dark as markings on body. Legs, including front and rear of thighs, pale straw yellow with scattered pale-brown flecks. Short, pale straw-yellow stripe on back surface of distal portion of shank and on heel. Irregular brown blotch dorsally on each wrist. Chin and throat evenly dusted with brown punctations except mid-ventrally on chin, where its absence forms a pale line, adjacent to which the brown dusting is more heavily concentrated; abdomen also heavily dusted with brown, but with more irregular distribution than on chin and throat. Palmar and plantar surfaces pale straw yellow evenly dusted with brown punctations. Iris dark brown.

*Measurements of holotype (in mm).*–SV = 20.1, TL = 10.0, HW = 8.0, HL = 7.3, IN = 2.2, EN = 1.7, SN = 2.9, EY = 2.9, TY = 1.0, 3rdF = 1.14, 4thT = 0.88, mass = 0.8 g.

#### Variation.

The female is larger than the males and has a slightly larger tympanum and greater disparity in disc widths between the fingers and toes (Table 3). It remains to be determined from a larger sample size whether these represent instances of sexual dimorphism. Otherwise, there is little mensural variation of interest in the small sample.

**Table 3.** Mensural data for the type series of *Oreoprhyne parkopanorum* sp. n. All measurements except mass are in mm.

**Character**	**BPBM 22787**	**BPBM 22788**	**BPBM 22789**	**BPBM 22790**	**PNGNM 24152**
Sex	subadult M	M	F	M	M
mass (g)	0.35	0.60	0.80	0.55	–
SV	15.8	17.5	20.1	17.7	17.5
TL	8.3	9.0	10.0	8.0	8.7
EN	1.4	1.7	1.7	1.6	1.6
IN	1.8	1.9	2.2	1.8	2.1
SN	2.5	2.7	2.9	2.5	2.4
TY	0.7	0.8	1.0	0.8	0.8
EY	2.2	2.5	2.9	2.7	2.5
HW	6.3	7.1	8.0	6.9	7.0
HL	5.6	6.3	7.3	6.2	6.3
3rdF	0.78	0.90	1.14	0.85	1.14
4thT	0.70	0.79	0.88	0.79	0.93
TL/SV	0.53	0.51	0.50	0.45	0.50
EN/SV	0.089	0.097	0.085	0.090	0.091
IN/SV	0.114	0.109	0.109	0.102	0.120
SN/SV	0.16	0.15	0.14	0.14	0.14
TY/SV	0.044	0.046	0.050	0.045	0.046
EY/SV	0.14	0.14	0.14	0.15	0.14
HW/SV	0.40	0.41	0.40	0.39	0.40
HL/SV	0.35	0.36	0.36	0.35	0.36
3rdF/SV	0.049	0.051	0.057	0.048	0.065
4thT/SV	0.044	0.045	0.044	0.045	0.053
EN/IN	0.78	0.89	0.77	0.89	0.76
3rdF/4thT	1.11	1.14	1.30	1.08	1.23
HL/HW	0.89	0.89	0.91	0.90	0.90

Snout profile varies from truncate to shallowly angulate when viewed from the side, shallowly angulate to acutely rounded when viewed from above. The female holotype is more heavily tuberculate than the male paratypes, which typically have the hourglass-shaped rows of tubercles well-defined dorsolaterally and also have scattered tubercles on the lateral surfaces, as well as smaller pustules apparent elsewhere, especially posterior to the tympanum.

The holotype is the only specimen with a broad vertebral stripe and heel stripe (Fig. 2D), but two males (BPBM 22790 and PNGNM 24152) have narrower, intermittent vertebral lines. All specimens have the mid-dorsal region paler than the sides, giving the impression of a paler hourglass-shaped region mid-dorsally. Most specimens are moderately heavily dusted with brown dorsally, as seen in the holotype, but the subadult male (BPBM 22787) is paler overall, with brown dusting less dense dorsally. This specimen also has two rows of dark-brown dashes laterally, extending from near forearm insertion to posterior third of body, the upper row at the level of the dark-brown supratympanic dash, the lower at the level of forearm insertion. BPBM 22790 also has these two rows of dark-brown lateral dashes well defined, but the other three specimens have brown flecking and spotting more irregularly distributed across the lateral surfaces. The snouts of all specimens are paler than the remainder of the head, but brown flecking occurs in this field in some specimens, thereby making the feature less obvious. The males all have an even dusting of brown punctations ventrally, as seen in the holotype, but also have large, darker-brown spots scattered across the ventrum, giving the impression of a pale venter with scattered large brown flecks; these spots are weaker in BPBM 22788 than in the other specimens. In the subadult male, these larger brown spots are arrayed more or less into two rows extending from the chin to the abdomen. None of the males has the pale, brown-bordered, mid-ventral line seen on the chin of the holotype.

#### Color in life.

Field notes for BPBM 22787 note: “Dorsum light yellow brown with narrow dark-brown lines. Fore and aft of thigh and rear of shank orange-red. Venter pale straw with two rows of dark-gray flecks on chin and throat. Iris light brown.” The holotype, BPBM 22789 (Fig. 2D) was similar but had a yellow stripe from chin to abdomen, another across the pectoral region, and an orange mid-dorsal stripe. Brown dorsolateral and postocular markings are more evident in some animals (Fig. 2C) than others (Fig. 2D). Animals are more orangish during the night and yellower during the day. The orange-red on the hidden surfaces of the thighs fades to pale straw in preservative.

#### Call.

The call is uncertain. I heard two undetermined frog calls at the type locality that are consistent with *Oreophryne* species from the north-coast ranges. One of these was a rattle call, the other was a series of high-pitched peeps. But I could associate neither call with a particular frog, so the identities of both are undetermined. One of them almost certainly represents *Oreoprhyne parkopanorum*, but I cannot say which.

#### Etymology.

The species name is a genetive plural honorific for the people of Parkop Village, whose unflagging help and friendliness made my expedition to the Torricelli Mts. successful and most pleasant.

#### Range.

Known only from the upper elevations of Mt. Sapau, Torricelli Mts., West Sepik Province, Papua New Guinea at an elevation of 1050–1320 m (Fig. 4, star). It probably occurs in similar habitat elsewhere in the Torricelli Mts. and may occur in the upper elevations of other nearby north-coast ranges.

#### Ecological notes.

This species inhabits primary mossy cloud forest at 1200–1300 m. We found our specimens active at night on moss-covered tree trunks from 6 cm to 2 m above ground. Forest in this area has a canopy of approximately 20 m height, many epiphytes, and a thick layer of leaf litter and duff.

Mature males were 17.5–17.7 mm in SV, but one male was still immature at 15.8 mm SV.

Syntopic frogs include *Albericus brunhildae*, *Austrochaperina septentrionalis*, *Choerophryne longirostris*, *Choerophryne rostellifer*, *Copiula tyleri*, *Hylarana jimiensis*, *Hylarana volkerjane*, *Hylophorbus* sp., *Liophryne schlaginhaufeni*, *Litoria modica*, *Litoria wollastoni*, *Nyctimystes pulcher*, and *Xenorhina arboricola*.

### Oreophryne gagneorum sp. n.

http://zoobank.org/496AF6FA-988F-4805-B8A6-A0515CC8981E

http://species-id.net/wiki/Oreophryne_gagneorum

Figs 2E, F , 6

#### Holotype.

BPBM 20542 (field tag FK 10121), adult female, collected by F. Kraus and local villagers on S slope of Mt. Rossel, 11.3555°S, 154.2246°E, 720 m, Rossel Island, Milne Bay Province, Papua New Guinea, 5 May 2004.

#### Paratypes

**(n = 52).** Same data as holotype (BPBM 20538–41, 20543–57, PNGNM 24153–55); same data as holotype, except collected 3 May (BPBM 20531–37), 6 May (BPBM 20558–60, PNGNM 24156–60), 7 May (BPBM 20561–69), 8 May (BPBM 20570), 9 May (BPBM 20571), and 10 May (BPBM 20572) 2004; halfway between 11.3354°S, 154.2223°E and 11.3354°S, 154.2247°E, 275–280 m (BPBM 43075, PNGNM 24161).

#### Diagnosis.

*Oreophryne gagneorum* can be distinguished from all congeners by its unique combination of small size (adult male SV = 16.3–20.0 mm, adult female SV = 19.0–23.5 mm); cartilaginous connection between the scapula and procoracoid; well-webbed toes; third and fifth toes subequal in length; steeply oblique lores; leg moderately long (TL/SV = 0.46–0.59); snout typically longer than broad (EN/IN = 1.00–1.33, EN/SV = 0.093–0.121, IN/SV = 0.080–0.109); head relatively broad (HW/SV = 0.36–0.43, HL/HW = 0.82–0.92); tympanum small (TY/SV = 0.034–0.051); finger and toe discs relatively broad (3rdF/SV = 0.063–0.086, 4thT/SV = 0.048–0.066, 3rdF/4thT = 1.18–1.47); shanks either unicolor or flecked/mottled with dark brown; pale-tan iris suffused or veined with black; and call a rapid series of short notes (23–190 ms) delivered at a rate of 9.57–11.32 notes/s with a dominant frequency of 3070–3510 Hz.

#### Comparisons with other species.

The new species differs from all other Papuan *Oreophryne* except *Oreophryne crucifer* and *Oreophryne kampeni* in its unique combination of having a cartilaginous connection between the scapula and procoracoid and having well-webbed toes. *Oreophryne gagneorum* differs from *Oreophryne crucifer* in its longer leg (TL/SV = 0.46–0.59 vs. 0.45 in *Oreophryne crucifer*), longer snout (EN/SV = 0.093–0.121 vs. 0.092 in *Oreophryne crucifer*), shorter head (HL/HW = 0.82–0.92 vs. 0.78 in *Oreophryne crucifer*), smaller tympanum (TY/SV = 0.034–0.051 vs. 0.054 in *Oreophryne crucifer*), relatively wider finger discs (3rdF/4thT = 1.18–1.47 vs. 1.13 in *Oreophryne crucifer*), fourth finger longer than the second (second longer than fourth in *Oreophryne crucifer*), and absence of a golden-yellow bar between the eyes. It differs from *Oreophryne kampeni* in having the third and fifth toes subequal in length (third distinctly longer in *Oreophryne kampeni*), more oblique loreal region (lores almost vertical in *Oreophryne kampeni*), usually longer leg (TL/SV = 0.46–0.59 vs. 0.44–0.47 in *Oreophryne kampeni*) and longer snout (EN/IN = 1.00–1.33 vs. 0.094–1.05 in *Oreophryne kampeni*), larger toe discs (4thT/SV = 0.048–0.066 vs. 0.042–0.048 in *Oreophryne kampeni*), and the shanks without brown spots (shanks conspicuously patterned with round dark-brown spots in *Oreophryne kampeni*).

*Oreophryne cameroni*, *Oreophryne idenburghensis*, *Oreophryne oviprotector*, and *Oreophryne waira* also have a cartilaginous connection between the scapula and procoracoid and webbing between the toes, but they differ from *Oreophryne gagneorum* in having basal instead of extensive toe webbing. Furthermore, *Oreophryne cameroni* has the fifth toe distinctly longer than the third, a shorter snout (EN/IN = 0.94–0.95 vs. 1.00–1.3 in *Oreophryne gagneorum*), dark-brown iris, and call consisting of more slowly delivered notes (2.64–2.75 notes/s vs. 9.57–11.32 notes/s in *Oreophryne gagneorum*) of lower dominant frequeny (2870–2940 Hz vs. 3070–3510 Hz in *Oreophryne gagneorum*). *Oreophryne idenburghensis* is a much larger species (SV = 43–47 mm vs. 16.3–23.5 mm in *Oreophryne gagneorum*), with the fifth toe distinctly longer than the third, and with a broader snout (EN/IN = 0.087–0.091 vs. 1.00–1.33 in *Oreophryne gagneorum*); *Oreophryne oviprotector* has the fifth toe distinctly longer than the third, is lime green dorsally with a green bar between the eyes and a white ring around the orbit, and has a rattle call; and *Oreophryne waira* is a slightly smaller species (SV = 17.8–21.0 mm vs. 16.3–23.5 mm in *Oreophryne gagneorum*) with a rattle call (vs. a high-pitched whinny in *Oreophryne gagneorum*) and a shorter snout (EN/IN = 0.094–0.105 vs. 1.00–1.33 in *Oreophryne gagneorum*). All other Papuan *Oreophryne* have either a ligamentous connection between the scapula and procoracoid, lack toe webbing entirely, or both.

#### Description of holotype.

**Figure 6.**
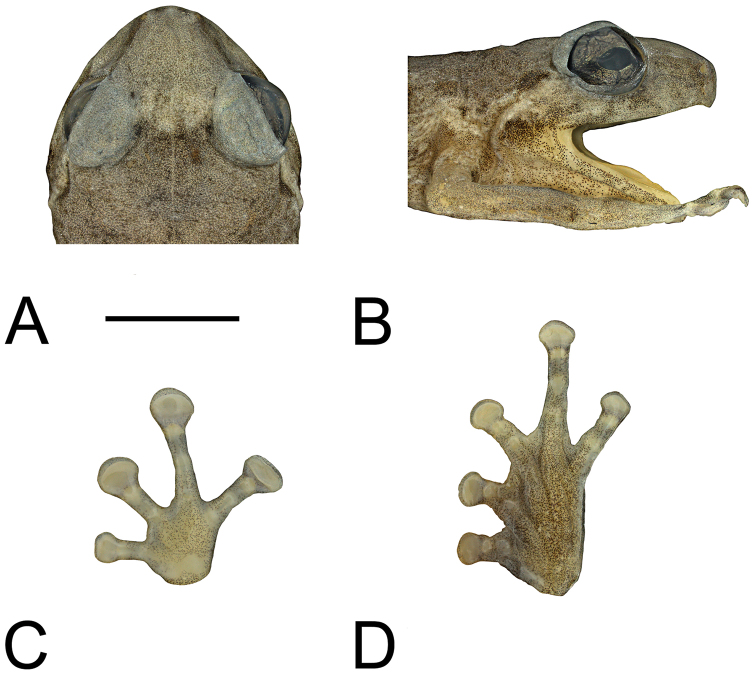
**A** Top of head **B** side of head **C** palmar view of left hand, and **D** plantar view of left foot of holotype of *Oreophryne gagneorum* sp. n. (BPBM 20542). Scale bar = 5 mm.

An adult female with an incision on right side and left pectoral region dissected. Procoracoid connected to the scapula by a narrow cartilaginous rod. Head wide (HW/SV = 0.40), with steeply oblique loreal region; upper lip inflated. Canthus rostralis rounded, straight when viewed from above (Fig. 6A). Nostrils directed laterally, much closer to tip of snout than to eyes. Internarial distance slightly wider than distance from naris to eye (EN/IN = 1.25, IN/SV = 0.085, EN/SV = 0.106). Snout truncate when viewed from the side (Fig. 6B), shallowly angulate when viewed from above (Fig. 6A). Eyes moderately large (EY/SV = 0.14); eyelid approximately two-thirds width of interorbital distance. Tympanum small (TY/SV = 0.034), but with a distinct annulus, partly covered by skin posterodorsally. Weak supratympanic fold present; another weak fold extends posteroventrally from rear margin of tympanum. Skin smooth above and below, except abdomen granular. Fingers unwebbed, bearing discs with terminal grooves; relative lengths 3>4>2>1 (Fig. 6C). Finger discs approximately twice width of penultimate phalanges except for F1, which is approximately 1.5 times as wide as penultimate phalanx (3rdF/SV = 0.074). Subarticular tubercles low but distinct; inner metacarpal tubercle a large, low oval; outer a low circle. Toes well webbed, formula **I** 2–2 **II** 2.7–3.6 **III** 3–4.5 **IV** 4.7–3.6 **V**; bearing discs with terminal grooves; relative lengths 4>5=3>2>1 (Fig. 6D). Toe discs smaller than those of fingers (4thT/SV = 0.060, 3rdF/4thT = 1.24), approximately 1.5 times width of penultimate phalanges. Subarticular tubercles low but distinct; inner metatarsal tubercle a narrow oval; outer not apparent. Hind legs rather long (TL/SV = 0.49).

In preservative, dorsum pale tan minutely speckled with brown, with a narrow, partially obscured tan vertebral line; limbs, including rear of thighs, same color as dorsum. Pale patch of lighter tan extends between eyes, narrowly margined posteriorly by dark brown. Short pale-cream dash extends from behind eye, through tympanum, to end near forearm insertion; this is bordered above and below by small, diffuse fields of brown. Lateral and ventrolateral surfaces suffused with pale cream. Venter pale straw yellow minutely stippled with brown, this more concentrated on chin and throat, somewhat sparser posteriorly; under limbs, hands, and feet stippled likewise. Sparse brown canthal stripe and subocular blotch present. Iris pale tan veined with black, which is especially concentrated in a horizontal plane before and behind the pupil.

*Measurements of holotype (in mm).*—SV = 23.5, TL = 11.6, HW = 9.5, HL = 8.3, IN = 2.0, EN = 2.5, SN = 3.5, EY = 3.2, TY = 0.8, 3rdF = 1.73, 4thT = 1.40, mass = 1.20 g.

#### Variation.

The only apparent sexual dimorphism in this species is in size; females are larger than males in both mass and SV (Table 4). Otherwise, standard deviation of variables largely accords with the size of the mensural character, and variation across most variables is tight (Table 4). The snout shape varies from truncate to slightly rounded in lateral view and from shallowly angulate to slightly rounded in dorsal view. The tympanum is usually partially embedded in the surrounding skin, giving the impression that it sits in a depression. Webbing between the toes is always well developed, as in the holotype, and never merely basal. In life, animals have obvious scattered tubercles (Fig. 2E, F), but these become obscure in preservative.

**Table 4.** Mensural variation among adults of *Oreophryne gagneorum* sp. n. Measurements are in mm, except for mass (g).

**Character**	**Males (n = 35)**			**Females (n = 14)**		
	**mean**	**SD**	**range**	**mean**	**SD**	**range**
mass	0.61	0.0168	0.40–0.85	0.86	0.0412	0.65–1.20
SV	18.5	0.1790	16.3–20.0	20.5	0.3410	19.0–23.5
TL	9.5	0.0754	8.5–10.5	10.6	0.1626	9.9–11.7
EN	2.0	0.0189	1.8–2.2	2.2	0.0359	2.0–2.5
IN	1.8	0.0185	1.5–1.9	1.9	0.0277	1.8–2.1
SN	2.9	0.0234	2.5–3.2	3.2	0.0633	2.8–3.5
TY	0.8	0.0134	0.6–0.9	0.9	0.0334	0.7–1.1
EY	2.6	0.0346	2.2–3.0	2.8	0.0518	2.5–3.2
HW	7.4	0.0825	6.5–8.2	8.2	0.1482	7.6–9.5
HL	6.5	0.0577	5.8–7.1	7.3	0.1475	6.6–8.3
3rdF	1.34	0.0187	1.13–1.56	1.52	0.0479	1.26–1.84
4thT	1.03	0.0146	0.86–1.23	1.15	0.0364	0.94–1.41
TL/SV	0.51	0.0049	0.46–0.59	0.52	0.0044	0.49–0.54
EN/SV	0.106	0.0008	0.093–0.117	0.105	0.0009	0.097–0.111
IN/SV	0.095	0.0009	0.80–0.104	0.093	0.0010	0.085–0.098
SN/SV	0.16	0.0013	0.14–0.17	0.15	0.0019	0.14–0.17
TY/SV	0.042	0.0007	0.035–0.051	0.043	0.0015	0.034–0.051
EY/SV	0.14	0.0013	0.12–0.16	0.14	0.0016	0.13–0.15
HW/SV	0.40	0.0026	0.36–0.43	0.40	0.0022	0.38–0.41
HL/SV	0.35	0.0018	0.32–0.37	0.36	0.0023	0.35–0.37
3rdF/SV	0.072	0.0007	0.065–0.082	0.074	0.0016	0.063–0.086
4thT/SV	0.055	0.0007	0.049–0.065	0.056	0.0011	0.048–0.066
EN/IN	1.12	0.0129	1.00–1.33	1.13	0.0162	1.00–1.25
3rdF/4thT	1.31	0.0122	1.18–1.47	1.33	0.0200	1.20–1.43
HL/HW	0.87	0.0044	0.82–0.92	0.89	0.0056	0.85–0.91

This species presents a diverse array of color patterns in brown and gray. The dorsum varies from pale tan to dark brown, and may be uniform in pattern but more often with ill-defined dark smudges or suffusions of dark color that frequently form a vague, paler hourglass pattern mid-dorsally and/or a poorly defined, dark scapular W. Occasionally, there will be a large orange-tan blotch mid-dorsally, usually on the posterior half of the dorsum; there are also orange-tan blotches on the heels of two specimens. The pale cream or tan postocular dash is always present and extends through the tympanum; this is invariably bordered above by a dark-brown dash followed by a brief hiatus and another short brown dash over the forearm insertion. There is typically a diffuse dark-brown field below the cream postocular stripe; occasionally this is better developed into another brown dash or blotch. The dark-brown canthal stripe and subocular blotch may be present, absent, or only vaguely suggested. Top of the snout is often, but not always, paler than the remainder of the dorsum; there is often either a dark-brown or pale-tan bar extending between the eyes, but these too are variably present. A narrow, pale-tan vertebral line is present in 25 of the specimens; this is often broken or developed only anteriorly. Lumbar ocelli are almost always absent and are poorly developed in the few specimens in which they occur. Ventral ground color is typically pale straw yellow with the overlying dark pigment varying from minute and evenly distributed stippling to dense evenly distributed stippling to dense, aggregated dark stippling. Consequently, the impression of ventral coloration to the naked eye varies from evenly pale brown to evenly dark brown to pale brown with dark-brown flecks. Four specimens have poorly defined, pale-straw lines mid-ventrally on the chin and throat, and another four have pale-gray flecks scattered across the belly. Iris color is always pale tan either suffused or veined with black, this is usually concentrated in a horizontal plane before and behind the pupil.

#### Color in life.

Field notes for BPBM 20531 in life recorded the color as: “Dorsum brown with darker brown mottling and tan stripe laterally, below which is dark brown. Iris tan. Tan postocular stripe. Venter pale gray stippled with dark gray. Rear of thighs dark brown.” BPBM 20532 was dark tan dorsally with a few dark-brown spots; the rear of thighs were the same as the dorsum. BPBM 20533 was also brown dorsally with vague brown markings, a pale tan postocular stripe, and a small amount of yellow in the groin. The rear of the thighs were brown with a few light-gray stipples. Chin to chest was dark gray with light-gray flecks, and the abdomen and undersides of the legs were light gray heavily flecked with dark gray. BPBM 20534 had a tan vertebral stripe and a dusky red patch in groin and front and rear of thighs. BPBM 20535 was chocolate brown dorsally with a cream postocular stripe, yellow in the inguinal region, and dusky brick red in groin and hidden surfaces of thighs. The holotype was pale tan-gray in life with a few, scattered red-brown spots (Fig. 2E); BPBM 20544 was dark brown with an orangish hourglass-shaped figure mid-dorsally, white-tipped tubercles, tan inter-ocular bar, and cream on the sides (Fig. 2F). Both of these animals exhibited silver irises with a reddish-brown horizontal bar through the pupil.

#### Call.

This species was the predominant frog calling around the summit of Mt. Rossel. I recorded 14 calls from six animals, and calls segregated into two types: a long and a short call (Table 5). The former was the most commonly produced call, with the shorter call being produced more frequently when conditions were drier. Note-delivery rate of both call types is so rapid that to the human ear calls sounds like a high-pitched whinny.

**Table 5.** Call data for six specimens of *Oreophryne gagneorum* sp. n. from Rossel Island, Milne Bay Province, PNG. Numbers for call parameters are mean ± SD (range).

**Specimen**	**Call type**	**Temperature (˚C)**	**Number of calls**	**Call duration (s)**	**Notes/ call**	**Note duration (s)**	**Inter-note duration (s)**	**Repetition rate (notes/s)**	**Dominant frequency (kHz)**
BPBM 20558	long	22.8	3	2.20 ± 0.1106<br/> (1.98–2.33)	21–24	0.045 ± 0.0028<br/> (0.033–0.157)	0.052 ± 0.0012<br/> (0.032–0.082)	10.46 ± 0.0887<br/> (10.30–10.61)	3.38 ± 0.0062<br/> (3.26–3.51)
BPBM 20559	long	22.8	2	2.20 ± 0.0650<br/> (2.13–2.26)	23–24	0.036 ± 0.0043<br/> (0.028–0.190)	0.060 ± 0.0010<br/> (0.051–0.078)	10.71 ± 0.0893<br/> (10.62–10.80)	3.32 ± 0.0059<br/> (3.19–3.37)
BPBM 20560	long	22.8	1	2.92	31	0.037 ± 0.0045<br/> (0.023–0.170)	0.060 ± 0.0018<br/> (0.036–0.079)	10.62	3.38 ± 0.0100<br/> (3.23–3.47)
BPBM 20571	long	22.2	2	2.45 ± 0.0900<br/> (2.36–2.54)	26–28	0.050 ± 0.0036<br/> (0.041–0.182)	0.041 ± 0.0014<br/> (0.029–0.067)	11.02 ± 0.0033<br/> (11.02–11.02)	3.27 ± 0.0054<br/> (3.21–3.31)
BPBM 20538	short	22.6	4	0.93 ± 0.0263<br/> (0.85–0.96)	9–10	0.059 ± 0.0023<br/> (0.047–0.105)	0.038 ± 0.0005<br/> (0.032–0.044)	10.25 ± 0.2285<br/> (9.57–10.59)	3.12 ± 0.0045<br/> (3.07–3.18)
BPBM 20572	short	22.1	2	1.03 ± 0.0350<br/> (0.99–1.06)	11–12	0.058 ± 0.0033<br/> (0.046–0.112)	0.036 ± 0.0011<br/> (0.026–0.043)	11.22 ± 0.1048<br/> (11.11–11.32)	3.46 ± 0.0104<br/> (3.30–3.50)

**Figure 7.**
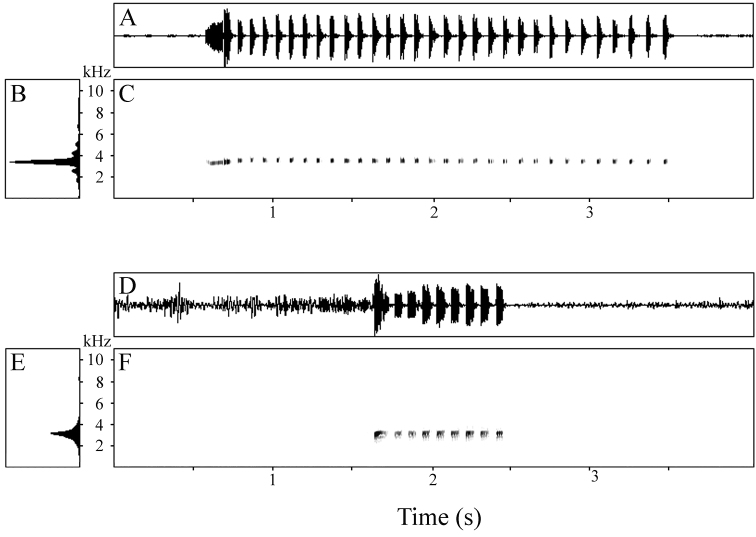
**A** Waveform **B** power spectrum, and **C** spectrogram of 31-note long call of paratype of *Oreophryne gagneorum* sp. n. (BPBM 20560) recorded on Mt. Rossel, Rossel Island, 6 May 2004, air temperature 22.8 °C, and **D** waveform **E** power spectrum, and **F** spectrogram of 9-note short call of paratype of *Oreophryne gagneorum* sp. n. (BPBM 20538), recorded on Mt. Rossel, Rossel Island, 5 May 2004, air temperature 22.6 °C.

The more commonly delivered, long calls (n = 8) ranged from 1.98–2.92 s in duration and consisted of a series of 21–31 notes emitted at a rate of 10.30–11.02 notes/s (Table 5). The first note of each call was much longer than the remainder (Fig. 7A), being 143–190 ms in length (mean 167 ms); subsequent notes were much briefer, with a mean of means of 37 ms (range 23–56 ms). The interval between notes was somewhat longer than the notes themselves, with a mean of means of 53 ms and range of 29–82 ms. The first note had a rounded amplitude envelope initially, followed by a short, sharp drop in volume, quickly succeeded by a large terminal spike (Fig. 7A); subsequent notes attained maximum volume rapidly and then decreased at an increasing rate, resulting usually in a concavely triangular amplitude envelope (Fig. 7A). Notes lacked harmonics, pulsing, and frequency modulation (Fig. 7C). The dominant frequency of calls varied within a very narrow window (Fig. 7B), with a mean of means of 3337 Hz and range of 3212–3514 Hz.

The less-frequently delivered short calls (n = 6) contained only 9–12 notes but were emitted at a rate similar to that found in the longer calls (9.57–11.32 notes/s); calls ranged from 0.85–1.06 s in duration (Table 5). Notes of these calls were not so internally divergent in length as those in the long calls. For each call, the first note was only approximately twice the length of the remainder (Fig. 7D), being 92–112 ms in length (mean 102 ms), compared to a mean of means of 54 ms (range 46–62 ms) for subsequent notes (Table 5). The interval between notes was shorter than in the long calls, with a mean of means of 37 ms and range of 26–44 ms. Hence, the length difference between notes and inter-note intervals was not as great as seen in the long calls. The first note attained maximum volume rapidly, decreased rapidly to a lower amplitude, and then maintained that until the end of the note (Fig. 7D); subsequent notes also increased to maximum amplitude quickly, maintained that volume rather evenly, and then decreased quickly to termination, producing an approximately square-shaped amplitude envelope (Fig. 7D). Notes lacked harmonics, pulsing, and frequency modulation (Fig. 7F). The dominant frequency of calls varied within a very narrow window (Fig. 7E), with a mean of means of 3289 Hz and range of 3068–3497 Hz.

#### Etymology.

The name is an honorific for Betsy and Wayne Gagné, dedicated and inspiring conservationists of Pacific island biotas and among the few western researchers to visit Mt. Rossel, being members of the 1979 Lae Forestry Institute botanical expedition to that mountain.

#### Range.

Endemic to Rossel Island, Milne Bay Province, PNG. It was very common along the upper elevations of Mt. Rossel at 720–750 m elevation, but I found it to occur as low as 280 m elevation.

#### Ecological notes.

The type locality consists of dense cloud forest on a steep ridge on the south slope of Mt. Rossel. Forest here is approximately 5–10 m high, and large gingers and tree ferns are common. Even when rainfall is absent moisture at this site is largely constant due to fog drip from clouds blowing over the ridge. Soil consists of mud on the slopes but with pockets of humus, especially along the ridge. The region is subject to major landslides, with a large landslide extending from just below the type locality to the bottom of an adjacent valley at approximately 250 m elevation. Animals were abundant at the type locality. They also occured less commonly in tall, lowland secondary forest growing at 280 m on clay mud and scree slides. In this area the undergrowth was not dense, and palms, pandanus, and ferns were common.

Frogs called from late afternoon through early morning at the type locality; calling perches were typically stems or leaves from 1–4 m above ground. Frogs typically emitted the longer advertisement calls when conditions were wet. In those circumstances they were not shy and were easily captured. Under drier conditions, the frogs gave slower calls at more erratic intervals, and they often called from hidden perches. Calls in the population would often move in a wave of chorusing activity across the mountain.

The smallest mature male was 16.3 mm SV, and it was recorded calling, but another male at 16.5 mm SV was not yet mature. The smallest mature female had a SV of 19.0 mm; two immature females were 17.0 and 17.4 mm long. Hence, males mature at a smaller size than do females.

The frog community on Rossel Island is rather depauperate; syntopic frogs include only *Austrochaperina yelaensis*, *Barygenys exsul*, *Cophixalus cupricarenus*, *Cophixalus kethuk*, an undescribed *Copiula*, *Litoria eschata*, *Litoria louisiadensis*, *Mantophryne louisiadensis*, *Nyctimystes perimetri*, and an undescribed *Oreophryne*.

## Discussion

The outlying mountain ranges that occur along the northern coast of New Guinea are derived from a series of offshore island arcs that have been sequentially accreted onto the New Guinea mainland in a west-to-east progression over the past 20 million years (Davies et al. 1997; Davies 2012). This region in composite may be referred to as the Northern Island-Arc Terranes (Pigram and Symonds 1991) and is separate in origin from the adjacent Vogelkop Composite Terrane (the “bird’s-head” region of New Guinea) to the west, which was sutured to New Guinea approximately 12 MYA (Pigram and Symonds 1991; Polhemus and Polhemus 1998). Although currently remaining as offshore islands, Yapen and Biak islands in the west and New Britain in the east are parts of the same Northern Island-Arc Terranes system (cf., map in Polhemus and Polhemus 1998); they just haven’t accreted to the mainland yet.

Seven species of *Oreophryne* are now known from the mainland north-coast ranges of the Northern Island-Arc Terranes: *Oreophryne biroi*, *Oreophryne cameroni*, *Oreophryne geislerorum*, *Oreophryne hypsiops*, *Oreophryne parkeri*, *Oreoprhyne parkopanorum*, and *Oreophryne wolterstorffi* (Zweifel et al. 2003; this study). On geologically allied offshore terranes, *Oreophryne brachypus* is restricted to New Britain (Zweifel et al. 2003), *Oreophryne kapisa* to Biak Island (Günther 2003b), and *Oreophryne asplenicola*, *Oreophryne pseudasplenicola*, and *Oreophryne waira* to Yapen Island (Günther 2003b). In the eastern portion of the Vogelkop Composite Terrane – immediately adjacent to the Northern Island-Arc Terranes but geologically independent of them – *Oreophryne atrigularis*, *Oreophryne clamata*, *Oreophryne sibilans*, and *Oreophryne unicolor* are known from the Wandammen Peninsula (Günther et al. 2001; Günther 2003a). As yet, none of the species described from the Northern Island-Arc Terranes system has been reported in the Vogelkop Composite Terrane, or vice versa.

As a biogeographically related community, the species of the Northern Island-Arc Terranes show interesting patterns of phenotypic variation in a few characters that may be useful in indicating phylogenetic relationships among them. Of the mainland species, *Oreophryne cameroni* and *Oreoprhyne parkopanorum* are the only north-coast species to have a cartilaginous connection between the procoracoid and scapula, a situation shared only with the three species endemic to Yapen Island in the west. However, one would expect additional species with this feature to surface once the large expanses of intervening terrain in Indonesian New Guinea are better surveyed. Most other Papuan *Oreophryne* with a cartilaginous connection occupy portions of the central cordillera, although *Oreophryne clamata* is known from the eastern portion of the Vogelkop Composite Terrane, *Oreophryne kampeni* is known only from the type locality near Port Moresby and *Oreophryne gagneorum* is restricted to Rossel Island. These latter two locations are part of the East Papuan Composite Terrane that comprises southeastern New Guinea and adjacent islands. The remaining species of the Northern Island-Arc Terranes and of the East Papuan Composite Terrane, whether occurring insularly or on the mainland, all have a ligamentous connection between these pectoral elements. As a hypothesis for future testing, it will be interesting to determine whether these *Oreophryne* from Yapen Island are in fact closely related to the two species described herein or whether they have independently acquired this pectoral feature. The preliminary phylogenetic tree for western *Oreophryne* obtained by Köhler and Günther (2008) suggests that the latter may be the case.

Similarly interesting is that all *Oreophryne* from the Northern Island-Arc Terranes for which data are available exhibit one of only two call types: either a series of unpulsed peeps or a pulsed rattle. The calls of *Oreophryne asplenicola*, *Oreophryne cameroni*, *Oreophryne hypsiops*, *Oreophryne parkeri*, and *Oreophryne pseudasplenicola* are a series of peeps; those of *Oreophryne biroi*, *Oreophryne brachypus*, *Oreophryne geislerorum*, *Oreophryne kapisa*, and *Oreophryne waira* are rattles. The calls of *Oreoprhyne parkopanorum* and *Oreophryne wolterstorffi* remain unknown. This pattern also holds true for the *Oreophryne* of the Vogelkop Composite Terrane: the calls of *Oreophryne sibilans* and *Oreophryne unicolor* are peeps, those of *Oreophryne atrigularis*, and *Oreophryne clamata* are rattles. Both call types also occur among *Oreophryne* species in the central cordillera, but call types there are more diverse and include calls not easily placed in either of the preceding two categories (Zweifel et al. 2005; Kraus and Allison 2009a). More interesting is that most *Oreophryne* species from the East Papuan Composite Terrane have calls that represent two additional call types: either the high-pitched, rapid whinny found in *Oreophryne gagneorum* and a number of other, current undescribed, species, or a short honk (F. Kraus, unpubl. data), although additional call types that do not fit into these primary groups also occur (Menzies 2006; Kraus and Allison 2009b; F. Kraus, unpubl. data). In no case have I encountered *Oreophryne* species in this region having peep calls, but *Oreophryne geislerorum* and at least one undescribed species from this region have rattle calls. It is perhaps informative of phylogenetic history that some of these diverse call types (e.g., peep call, whinny call, honk call) should be exclusive to areas of New Guinea having very different geological histories. It remains to be determined whether call types will provide a better indicator of phylogenetic propinquity than several of the morphological features used for species discrimination. Because most of the species from southeastern New Guinea remain undescribed, I will explore this issue in greater detail upon their description.

In contrast to these characters, variation in toe webbing and relative length of third and fifth toes does not appear to divide into geographically discrete patterns. Species without toe webbing are largely, but not entirely, confined to the central cordillera and islands to the west, whereas those with either basal webbing or well-webbed toes are found throughout the region. And all variants in relative toe length are found throughout the region. Given that most *Oreophryne* species seem to have narrowly circumscribed geographic ranges suggestive of limited dispersal ability, the distribution patterns of these phenotypic features are consistent with independent origins of each character state.

Although the description of the new species treated herein now brings to seven the number of *Oreophryne* species reported from the north-coast region of New Guinea, the presence from these areas of additional specimens of uncertain identity (Zweifel et al. 2003) suggests that additional species likely await description. Furthermore, the large expanse of unexplored north-coast mountains in adjacent Papua Province of Indonesia will certainly disclose new species once they become more thoroughly investigated. Similarly, the description of *Oreophryne gagneorum* brings to eight the number of *Oreophryne* species described from the East Papuan Composite Terrane system (Parker 1934; Menzies 2006; Kraus and Allison 2009b). However, I have at least a dozen more new *Oreophryne* species remaining to be described from this region, and large portions of this terrane system remain unsurveyed. Hence, interpreting patterns of phenotypic variation in that region is premature at this time. But it is clear that the number of Papuan species contained within *Oreophryne* remains only poorly approximated.

## Supplementary Material

XML Treatment for Oreophryne cameroni

XML Treatment for Oreophryne parkopanorum

XML Treatment for Oreophryne gagneorum

## Appendix

### Specimens examined of Papuan *Oreophryne* with cartilaginous connection between procoracoid and scapula

*Oreophryne anamiatoi* (n = 20): Papua New Guinea: Southern Highlands Province: E slope Mt. Itukua, 5.6695°S, 142.6233°E, 2177 m (BPBM 33768, holotype; 33763–67, 33769–72, PNGNM 24097–99, paratypes), E slope Mt. Paramo, 5.6451°S, 142.6362°E, 1874 m (BPBM 33773–79, PNGNM 24100, paratypes).

*Oreophryne crucifer* (n = 1): Indonesia: Papua Province: Went Mts. (ZMA 5819, syntype).

*Oreophryne flava* (n = 1): Indonesia: Papua Province: Lorentz River, Kloofbivak (ZMA 5823, holotype).

*Oreophryne idenburghensis* (n = 2): Indonesia: Papua Province: 18 km SW Bernhard Camp, Idenburg River, 2150 m (AMNH 49663, holotype, AMNH 49666, paratype).

*Oreophryne kampeni* (n = 12): Papua New Guinea: Central Province: Moroka (BMNH 1947.2.12.14, holotype, BMNH 1947.2.12.43–44, paratypes, MSNG 29127, nine paratypes under the same number).

*Oreophryne notata* (n = 35): Papua New Guinea: Southern Highlands Province: E slope Mt. Itukua, Muller Range, 2170 m (BPBM 33672–706).
